# Acute Response of Circulating Vascular Regulating MicroRNAs during and after High-Intensity and High-Volume Cycling in Children

**DOI:** 10.3389/fphys.2016.00092

**Published:** 2016-03-14

**Authors:** Yvonne Kilian, Udo F. Wehmeier, Patrick Wahl, Joachim Mester, Thomas Hilberg, Billy Sperlich

**Affiliations:** ^1^Institute of Training Science and Sport Informatics, German Sport University CologneCologne, Germany; ^2^The German Research Centre of Elite Sport, German Sport University CologneCologne, Germany; ^3^Department of Sports Medicine, University WuppertalWuppertal, Germany; ^4^Departement of Molecular and Cellular Sport Medicine, Institute of Cardiovascular Research and Sport Medicine, German Sport University CologneCologne, Germany; ^5^Integrative and Experimental Exercise Science, Department of Sport Science, University of WürzburgWürzburg, Germany

**Keywords:** children, endurance, exercise, microRNAs, training adaptation

## Abstract

**Aim:** The aim of the present study was to analyze the response of vascular circulating microRNAs (miRNAs; miR-16, miR-21, miR-126) and the VEGF mRNA following an acute bout of HIIT and HVT in children.

**Methods:**Twelve healthy competitive young male cyclists (14.4 ± 0.8 years; 57.9 ± 9.4 ml·min^−1^·kg^−1^ peak oxygen uptake) performed one session of high intensity 4 × 4 min intervals (HIIT) at 90–95% peak power output (PPO), each interval separated by 3 min of active recovery, and one high volume session (HVT) consisting of a constant load exercise for 90 min at 60% PPO. Capillary blood from the earlobe was collected under resting conditions, during exercise (d1 = 20 min, d2 = 30 min, d3 = 60 min), and 0, 30, 60, 180 min after the exercise to determine miR-16, -21, -126, and VEGF mRNA.

**Results:** HVT significantly increased miR-16 and miR-126 during and after the exercise compared to pre-values, whereas HIIT showed no significant influence on the miRNAs compared to pre-values. VEGF mRNA significantly increased during and after HIIT (d1, 30′, 60′, 180′) and HVT (d3, 0′, 60′).

**Conclusion:** Results of the present investigation suggest a volume dependent exercise regulation of vascular regulating miRNAs (miR-16, miR-21, miR-126) in children. In line with previous data, our data show that acute exercise can alter circulating miRNAs profiles that might be used as novel biomarkers to monitor acute and chronic changes due to exercise in various tissues.

## Introduction

High-intensity interval training (HIIT) when compared to low-intensity high volume training (HVT) has been shown to induce similar physiological adaptations. Among adult athletes, numerous studies involving sophisticated invasive methods have identified the molecular and cellular events related to HIIT (Gibala, 2009; Gibala et al., 2009). However, from an ethical standpoint, invasive methodologies may not be appropriate in youth populations. Therefore, minimally invasive methods for assessing molecular and cellular events are needed to better understand important mechanisms of gene regulation and cellular adaptations following training among adolescent populations. Studies on HIIT and HVT with highly-trained teenage athletes have been conducted in triathlon (age: 15.8 ± 1.8 years; Wahl et al., 2013; Zinner et al., 2014), football (age: 13.5 ± 0.4 years; Sperlich et al., 2011), swimming (age: 16.6 ± 1.4 years; Faude et al., 2008), alpine-skiing (age: 17.4 ± 1.1 years; Breil et al., 2010), and cross-country-skiing (age: 17.5 ± 0.4 years; Sandbakk et al., 2013). However, these studies primarily focus on performance, with little attention given to hormonal responses following different exercise regimes (Engel et al., 2014; Zinner et al., 2014). Nevertheless, more cellular analyses like microRNAs (miRNAs) have not been investigated in children/adolescents yet. Up to now, only one study examined circulating miRNAs in young untrained men (age: 21.5 ± 4.5 years) after a single bout of steady-state cycling (70% VO_2max_; Aoi et al., 2013).

Recently, a novel, minimally invasive method for the analysis of miRNAs from 20 μL of capillary blood from the earlobe (Wehmeier and Hilberg, 2014) has been developed which enables assessment of stress-related changes at the molecular and cellular level, both in adults and children. miRNAs are small non-coding RNA molecules (containing about 18–22 nucleotides), which are involved in the regulation and control of the gene expression at the post-transcriptional level of approximately one-third of the human genes (He and Hannon, 2004; Lewis et al., 2005). Recent studies show, that extracellular miRNAs are detectable in a variety of biological fluids such as serum, plasma, urine, and saliva at rest. Furthermore, expression levels of extracellular miRNAs change in response to variable conditions such as physical exercise (Baggish et al., 2011, 2014), inflammation (Davidson-Moncada et al., 2010), and muscle hypertrophy (Davidsen et al., 2011). The circulating miRNAs (c-miRNAs) are present in a highly stable, cell-free form, and are protected by various mechanisms from endogenous RNase degradation and can be used as minimally invasive biomarkers in the blood (Fichtlscherer et al., 2011; Zampetaki et al., 2011). The exact mechanisms for the release and transport of cell-free miRNAs have yet to be investigated. There is however, initial evidence that miRNAs could be transported in exosomes, microvesicles, microparticles, lipid vesicles, or apoptotic bodies, and can be taken up by recipient cells exerting regulatory processes (Arroyo et al., 2011; Fichtlscherer et al., 2011).

Recent studies show that miRNAs play an important role in the control of angiogenesis and vascular integrity (Urbich et al., 2008). A number of miRNAs have since been described that are expressed at high levels in the endothelium and that regulate key genes and activities. Several studies showed that miR-16, -21, and -126 are important regulators of angiogenic processes, including the survival, maintenance, and formation of new capillaries (Urbich et al., 2008; Wang et al., 2008; Fish and Srivastava, 2009; Suárez and Sessa, 2009). These miRNAs—also called “angiomiR”—are highly expressed in endothelial cells, and have been shown to be regulated by exercise (Suárez and Sessa, 2009; Quintavalle et al., 2011; Fernandes et al., 2012). Recent reviews by Xu et al. (2015) and Altana et al. (2015) summarize numerous studies examining the response of (circulating) miRNAs to exercise. For circulating miRNA-126, the maximum expression level was determined during endurance exercise 30 min after the start (Uhlemann et al., 2014). Fernandes et al. (2012) have analyzed the expression of miR-16, -21, and -126 in muscles after exercise and reported an up-regulation of miR-21 and miR-126. However, researchers have yet to determine if circulating miRNAs change in children in response to exercise and if the molecular response is affected by different training protocols (Altana et al., 2015; Xu et al., 2015). HIIT or HVT, are well-known stimuli with positive effects on endothelial function and angiogenic processes (Egginton, 2009). However, the exact regulation remains unknown, especially in children. Previous studies demonstrated that levels of angiogenic growth factors in the circulation are influenced by training intensity and volume (Wahl et al., 2011, 2014). In a previous study, we discovered that endurance exercise—independent of intensity—promoted a phosphatidylserin-dependent uptake of endothelial microparticles (EMP) into target endothelial cells, which was associated with a protection of target cells against apoptosis (Wahl et al., 2014). Importantly, it has been shown that EMPs promote vascular endothelial repair by delivering functional miR-126 into recipient cells (Jansen et al., 2013).

Limited empirical attention has been devoted to angiogenic processes or capillarization in youth and adults. Investigations with children are entirely missing. Studies with adults demonstrate that the skeletal muscle capillarization under resting conditions, as well as the vascular endothelial growth factor (VEGF) mRNA expression and VEGF protein concentration in the circulation are lower among older adults (65 years) compared with young men (21 years) regardless of rest or after 45 min cycling at 50% VO_2max_ (Ryan et al., 2006). Furthermore, irrespective of the training status older men show fewer muscle capillary contacts of type IIA and IIX fibers compared to young men, although no difference in muscle capillarization of type I fibers exists (Proctor et al., 1995). Although, these studies did not compare adults with children, they provided initial indication that angiogenic processes might differ as a consequence of age. Hence, it seems logical that angiogenic processes might be different in a youth population compared to an older sample.

Given (1) the dearth of knowledge regarding responses to vascular regulating factors of children to HIIT and HVT, and (2) new developments in ethically justifiable methods for the analysis of miRNAs (Wehmeier and Hilberg, 2014), the aim of the present study was to specifically analyze the response of circulating miRNAs (miR-16, miR-21, miR-126) and VEGF mRNA to an acute bout of HIIT and HVT in children.

## Materials and methods

### Subjects and ethics statement

A total of 12 healthy competitive young male cyclists took part in this study (mean ± *SD* age: 14.4 ± 0.8 years, body mass: 57.1 ± 13.4 kg, size: 173.3 ± 11.4 cm; tanner stages: 3.6 ± 1.0, relative peak oxygen uptake (VO_2peak_): 57.9 ± 9.4 ml·min^−1^·kg^−1^). The participants had a training history of 3.7 ± 0.9 years. The training workload summed up to 100–150 km per week and consisted of continuous endurance training as well as HIIT (4 × 4 min, 30–30 s). Furthermore, the athletes regularly participated in cycling competitions on weekends, which are characterized by an intermittent exercise profile. The study protocol was performed in accordance with the declaration of Helsinki and approved by the ethical Committee of the German Sport University Cologne, Germany. The participants and their guardians were informed about the design, potential risks and benefits of the study, and gave written informed consent to participate in the study.

### Exercise study protocol

For the measurement of peak oxygen uptake (VO_2peak_) and the proper adjustments of training intensity, athletes performed an incremental step test on a cycle ergometer (Schoberer Rad Meßtechnik SRM GmbH, Juelich, Germany). The step test consisted of cycling at ≥ 80 rpm with an initial workload of 80 and 20 W increments every 3 min until volitional exhaustion. During each step, oxygen uptake (VO_2_; Cortex Metamax® 3B, Cortex Biophysik, Leipzig, Germany), heart rate (HR; Polar S710, Polar Electro GmbH, Büttleborn, Germany), and capillary lactate concentrations [La] (EKF Diagnostic Sales, Magdeburg, Germany) were determined.

**Figure 1:**
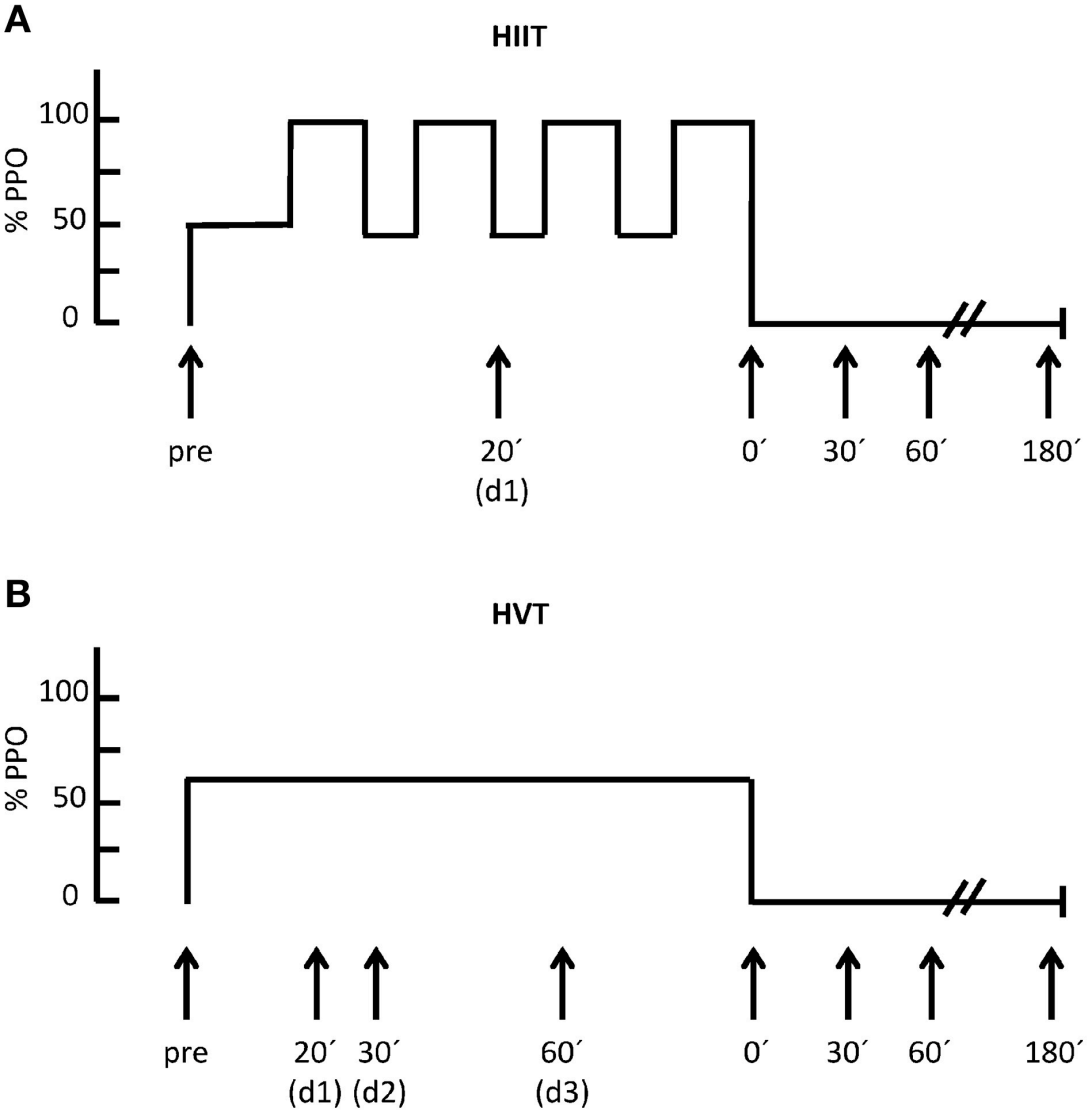
**Exercise study protocols for HIIT (A) and for HVT (B) including the duration, time points of blood sampling as well as the % of peak power output (%PPO); d = during**.

In order to test the effects of different endurance exercise protocols on the expression of miRNAs, the subjects visited the laboratory on two different days in a randomly chosen order. Between the first and second exercise protocol there were 7–9 days in which the participants continued their normal training. Prior to all testing, the cyclists were not allowed to perform strenuous exercise 48 h prior to testing. The HIIT session involved 4 × 4 min at 90–95% peak power output (PPO) separated by 3 min of active recovery (45% PPO) and a warm up for 10 min at 50% PPO. The HVT session involved 90 min at 60% PPO (Figure 1). All tests were carried out at the same time of day with identical ambient conditions (temperature: 23 ± 1°C; relative humidity: 35 ± 2%). During each session, spirometric data and heart rate were recorded continuously. Blood pressure was measured before and at the end of each intervention (Philips C3 patient monitor, Philips Deutschland Gmbh, Hamburg, Deutschland). Furthermore, Borg's Rating of Perceived Exertion (RPE) Scale was used to assess subjective perception of effort. A standardized food intake was given 2 h before and 30 min after each intervention. The subjects received 10 g carbohydrate and 0.25 g protein per kg bodyweight in the form of low fat chocolate milk and energy-bars as advised elsewhere (Pritchett et al., 2009).

### Measurements

#### Blood sampling

During each session, capillary blood samples (20 μL) were taken from the earlobe for the analyses of lactate [La] (EBIOplus, EKF Diagnostic Sales, Magdeburg, Germany) and pH (AVL Omni 6; Roche Diagnostics GmbH, Mannheim, Germany). During HIIT, blood was sampled for [La] at baseline (pre), post warm-up, after each 4 min bout, immediately after HIIT (post), and 5 min post. Blood for pH analysis was sampled prior to and after the second 4 min bout, as well as immediately post-HIIT. During HVT blood was sampled at pre, 15, 30, 60 min, post-HVT, 5 min post for [La] analysis and pre, 45 min, post-HVT for the determination of pH.

According to previous studies (Nielsen et al., 2010; Banzet et al., 2013; Tonevitsky et al., 2013) the analyses of miRNAs and the VEGF mRNA capillary blood samples were collected from the earlobe at the following time points: before (pre), during (d1 = 20 min, d2 = 30 min, d3 = 60 min), and 0 min (0′), 30 min (30′), 60 min (60′), and 180 min (180′) after each intervention (Figure 1).

#### Quantification of miRNA expression

The quantification of miRNA expression was performed following procedures outlined by Baggish et al. (2011) and explained here briefly as follows: for the quantification of mRNA and miRNA by RT-PCR, capillary blood was collected from the earlobe using a 20 μL capillary (End-to-End Kalium-EDTA capillaries, Sarstedt, Nürmbrecht, Germany). Wehmeier and Hilberg (2014) have shown, that the expression levels of mRNAs and miRNAs using either capillary blood or venous blood sample are nearly identical making the capillary miRNA analysis an appropriate method. Also Robison et al. (2009) demonstrated, that for the isolation of RNA capillary blood by fingerstick is comparable to the standard method of RNA isolation of whole blood by venipuncture. Furthermore, Wang et al. (2011) showed that the relative increase and changes over time of miRNAs (miR-133, -328) in plasma and whole blood are similar, although the absolute values in whole blood are higher. In fact, the use of capillary blood has one major advantage as the sample was mixed immediately with Trizol solution, so that all cellular processes were stopped immediately. The capillaries were transferred into a 1 mL peqGOLD TriFast reagent (peqLAB Biotechnologie, GmbH, Erlangen, Germany) and stored at −80°C. The isolation of total RNA from the capillary blood was performed according to the protocol for the peqGOLD TriFast reagent. According to Zhu and Altmann (2005) 18S rRNA can be regarded as an endogenous stable reference gene for real time PCR when using a co-application reverse transcription (Co-RT) method. Therefore the quantitative real time RT-PCR reaction was performed in a Stepone real time PCR unit (Applied Biosystems, Life Technologies, Darmstadt) running the two reactions of 18S rRNA and VEGF mRNA in the same tube, guaranteeing the same conditions (efficiency). The reaction mixture (20 μL) contained 0.16 μL RT Enzyme Mix, 10 μL RT-Mix, 100 nM of each primer and 2 μL sample. Each probe was tested in a duplet. To normalize differences in total RNA, the 18S ribosomal RNA was analyzed as a control by applying the primer TaqMan Ribosomal RNA Control Reagents VIC Probe (Applied Biosystems, Life Technologies, Darmstadt). The 5′ forward primers VEGF-AGGAGGAGGGCAGAATCATCA-3′, reverse 5′-VEGF TCTCGATTGGATGGCAGTAGC-3′, and the Taqman probe: 5′ (FAM)-TGGT GAAGTTCATGGATGTCTATCAGCGC-(TAMRA)-3′ were applied for the analysis of the VEGF mRNA (Yasuda et al., 2004). A dilution ladder of human standard RNA (Applied Biosystems, Life Technologies, Darmstadt) was applied to create a standard curve for quantitative analysis of the target RNAs. In addition, a standard curve for the 18S rRNA was measured to control the efficacy of the measurements. In all measurements the 18S rRNA was stable. The miRNAs-16, -21, -126 were determined using TaqMan microRNA assays (Life Technologies, Darmstadt, hsa-miR-16, hsa-miR-21, hsa-miR-126). A total of 5 μl of the isolated total RNA were used for the reverse transcriptase and thereof 1 μL for the real time PCR detection assay. The cycle thresholds (C_T_) data were determined using default threshold settings and the mean C_T_ was determined from duplicate PCRs. The relative levels of mRNA, e.g., miRNA expression were normalized to the endogenous control gene (18S rRNA) and were calculated using the 2$2^{-\Delta \Delta C}{}^{{}_{\text{T}}}$ method (Livak and Schmittgen, 2001). ΔC_T_ was calculated by subtracting the C_T_-values of 18S rRNA from the average C_T_-values of the target mRNA, e.g., miRNA. ΔC_T_-values were then compared (ΔΔC_T_) with each participant's own resting baseline at the pre-time point (normalized to fold change of one).

### Calculations

Total energy expenditure (EE) and total work were determined for each session. Total EE was considered as the sum of EE during warm-up (EE_WU_), EE during the intervals (EE_Int_), and EE during the recovery periods (EE_Rec_). For each of the periods, EE was calculated separately according to previous reports (Scott et al., 2006) based on oxygen consumption and lactate production (Δlactate):

$$\begin{array}{l} \text{E}{\text{E}}_{\text{WU}}:\text{time}\cdot 21.2\cdot \text{V}{\text{O}}_{2\text{WU}}+\Delta \mathrm{lactate}\cdot \mathrm{body}\;\mathrm{weight}\cdot \\ \;0.003\cdot 21.2\\ \text{E}{\text{E}}_{\text{Int}}\;:\text{time}\cdot 21.1\cdot \text{V}{\text{O}}_{2\text{Int}}+\mathrm{\Delta lactate}\cdot \mathrm{body}\;\mathrm{weight}\cdot \\ \;0.003\cdot 21.1\\ \text{E}{\text{E}}_{\text{Rec}}\;:\text{time}\cdot \left(19.6\cdot \left(\text{V}{\text{O}}_{2\text{Rec}}-VO_{2\mathrm{WU}}\right)+21.1\cdot VO_{2\mathrm{WU}}\right) \end{array}$$

During warm-up and the intervals, a caloric equivalent of 21.1 kJ per 1 l oxygen was used. For the recovery periods, the caloric equivalent of the excess post-exercise oxygen consumption was set at 19.6 kJ per 1 l oxygen (Scott, 2005; Scott and Kemp, 2005). An increase in 1 mmol · L^−1^ of lactate was considered equivalent to 3 ml of oxygen per kg body weight (Di Prampero and Ferretti, 1999). For WU, Δlactate was defined as the difference between after WU- and rest-values, and for the intervals, Δlactate was equivalent to the difference between the values after the first interval and after WU. The first interval was considered representative for all intervals as the power output was constant for all four intervals. Total work [kJ] was calculated by the following formula:

$$\begin{array}{l} \text{Total work}=\left(\text{MPO}\left[\text{W}\right]\cdot \text{exercise time}\left[\text{s}\right]\right)\cdot 1000^{-1}. \end{array}$$

### Statistical analyses

Statistical analyses of the data were performed using a statistics software package (Statistica for Windows, 7.0, Statsoft, Tulsa, OK; SPSS, Chicago, IL). Subject characteristics and exercise testing data are presented as means ± standard deviation (SD). miRNA and VEGF mRNA are presented as means ± standard error of the mean (SEM). All data were tested for normality with no further transformation needed. To test for differences, we used a Two-way repeated-measures ANOVA [intervention (HIIT, HVT); time (pre, d1, d2, d3, 0′, 30′, 60′, 180′)] with a Fisher *post-hoc* test. Correlation analyses were performed using the Spearman or Pearson's method as appropriate for the data distribution. The area under the curve (AUC) was calculated to compare the post-exercise recovery time points between HIIT and HVT. A Student's *t*-test was performed to test differences for the AUC between HIIT and HVT. Statistical differences were considered to be significant for *p* ≤ 0.05.

## Results

Mean power output, mean and peak lactate, mean and min pH, total work, energy expenditure, total oxygen consumption, mean and peak heart rate, and RPE for HIIT and HVT are shown in Table 1. Total work, total EE, and total oxygen consumption were higher during and after HVT (all *p* < 0.0001), whereas mean power output (*p* = 0.02), mean and peak heart rate, RPE, and systolic and diastolic blood pressure were higher in HIIT (all *p* < 0.0001). Mean and min pH was significantly lower (*p* = 0.006 – 0.002), whereas mean and peak [La] was higher (*p* < 0.0001) during HIIT compared to HVT.

**Table 1:** **Overview of the two interventions**.

	**High-intensity interval training**	**High-volume training**
Total time [min]	4 × 4 with 3 min recovery (=25 min)	90
% of peak power output	90–95	60
Mean power output [W]	163±39^*^	146±35
Mean heart rate [bpm]	189±11^*^	159±12
Peak heart rate [bpm]	194±10^*^	163±12
Mean lactate concentration [mmol·L^−1^]	5.3±2.2^*^	1.1±0.5
Peak lactate concentration [mmol·L^−1^]	6.3±2.7^*^	1.5±0.6
Mean pH [AU]	7.35±0.04^*^	7.39±0.02
Minimum ph [AU]	7.31±0.06^*^	7.38±0.02
Total work [kJ]	352±84^*^	786±187
Energy expenditure [kJ]	1866±525^*^	4172±1150
Total oxygen consumption [L]	83±24^*^	197±55
Ratings of perceived exertion [AU]	16±2^*^	12±2
Blood pressure systolic [mm Hg]	144±24^*^	118±19
Blood pressure diastolic [mm Hg]	89±17^*^	78±15

*Data are shown as mean ± SD*.

* *significantly different from high-volume training; p < 0.05*.

### mir-16

**Figure 2:**
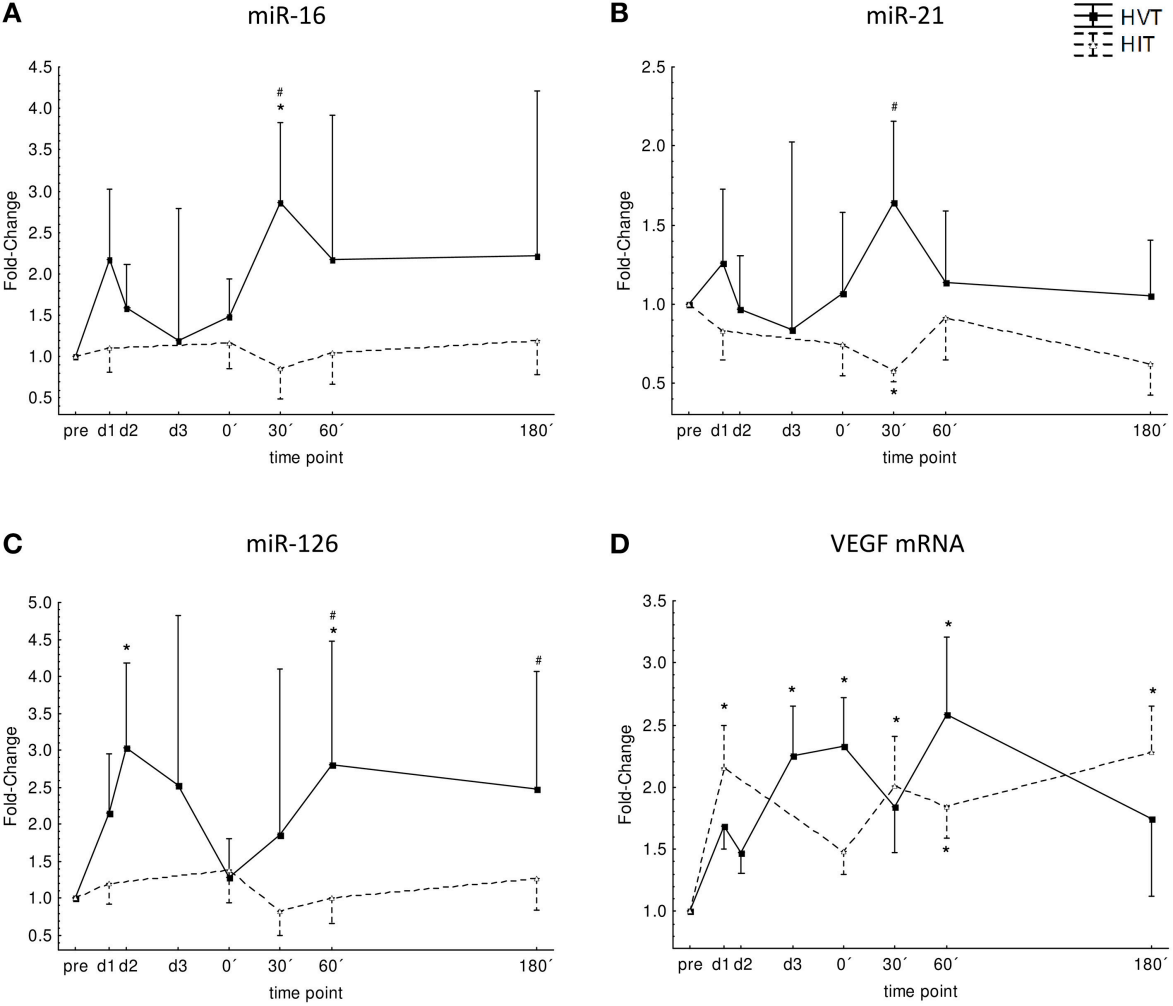
**Normalized expression level for miR-16 (A), miR-21 (B), miR-126 (C) and VEGF mRNA (D) before (pre), during (d1, d2, d3), and after (0′, 30′, 60′, 180′) each intervention HIIT (triangles, broken line), and HVT (squares, solid line)**. ^*^Significant different compared to pre-values of the same intervention (*p* < 0.05); ^#^significant different to HIIT (*p* < 0.05). Values are presented as means ± SE.

Over-all ANOVA showed no time effect (*p* = 0.46), no intervention effect (*p* = 0.14), and no interaction effect (intervention^*^time; *p* = 0.38). Post-hoc analysis revealed significantly higher levels 30′ post-exercise for HVT compared to HIIT (*p* = 0.008) and to pre-values (*p* = 0.02). The AUC for the post-exercise time points did not differ significantly between HIIT and HVT (193 ± 160 vs. 404 ± 282; Figure 2A).

### miR-21

Over-all ANOVA showed no time effect (*p* = 0.82), no intervention effect (*p* = 0.19), and no interaction effect (intervention^*^time; *p* = 0.23). Post-hoc analysis revealed significantly higher levels 30′ post-exercise for HVT compared to HIIT (*p* < 0.001) and significantly lower levels for HIIT 30′ post-exercise compared to pre-values (*p* = 0.02). The AUC for the post-exercise time points did not differ significantly between HIIT and HVT (134 ± 89 vs. 213 ± 152; Figure 2B).

### miR-126

Over-all ANOVA showed a time effect (*p* = 0.04), no intervention effect (*p* = 0.18), and no interaction effect (intervention^*^time; *p* = 0.16). Post-hoc analysis revealed significant increases at d2 (*p* = 0.01) and 60′ post-exercise (*p* = 0.02) for HVT as well as significant differences between HIIT and HVT for 60′ (*p* = 0.003) and 180′ (*p* = 0.04) post-exercise with higher values for HVT. The AUC for the post-exercise time points did not differ significantly between HIIT and HVT (197 ± 157 vs. 433 ± 316; Figure 2C).

No significant correlations were found between VO_2max_ and peak levels of miR-16 (HIIT: r = −0.41; HVT: *r* = 0.04), miR-21 (HIIT: *r* = 0.12; HVT: *r* = 0.31), and miR-126 (HIIT: r = −0.25; HVT: *r* = 0.41).

### VEGF mRNA

Over-all ANOVA showed a time effect (*p* = 0.02), no intervention effect (*p* = 0.86) and no interaction effect (intervention^*^time; *p* = 0.24). Post-hoc analysis revealed that VEGF mRNA significantly increased at d1 and 30′, 60′, and 180′ after HIIT (*p* < 0.003) and at d3, 0′ and 60′ after HVT (*p* < 0.05) when compared to baseline values. The AUC for the post-exercise time points did not differ significantly between HIIT and HVT (358 ± 132 vs. 388 ± 205; Figure 2D).

## Discussion

Altana et al. (2015) and Xu et al. (2015), recently summarized the few studies that have examined the impact of exercise in vivo on circulating miRNAs. As no such data was available in children, the aim was to investigate the acute responses of circulating angiogenic regulating miRNAs during and after high-intensity and high-volume cycling in youth. The present study showed that the miR-16, -21, -126 are upregulated during and after HVT. In contrast, the HIIT session did not affect the miRNA expression. The VEGF mRNA was up-regulated during and after both interventions.

miR-16 has been shown to be produced by human endothelial cells and is implicated in suppressing VEGF, VEGF receptor 2 (VEGFR2), basic fibroblast growth factor (bFGF), and fibroblast growth factor receptor 1 (FGF-R1; Chamorro-Jorganes et al., 2011; Triozzi et al., 2012). miR-16 reduces proliferation, migration, and angiogenic capacity of endothelial cells in vitro (Caporali and Emanueli, 2011). In young men, Aoi et al. (2013) reported that circulating miR-16 was affected neither by acute nor chronic exercise (Aoi et al., 2013). In the present study, childrens' response of miR-16 showed slight increases directly after commencing HVT, with a significant upregulation 30 min post-exercise. Conversely, HIIT did not influence the miR-16 expression level. In contrast with these findings, previous research with untrained adults (VO_2max_ 42.0 ± 2.0 mL·kg^−1^·min^−1^) showed a significant down-regulation of miR-16 (1.23-fold change) after a high intensity interval session (10 × 2 min at 76% VO_2peak_; Radom-Aizik et al., 2010).

In terms of angiogenesis, miR-21 induces angiogenic processes and the differentiation of endothelial stem cells through targeting phosphatase and tensin homolog (PTEN). This targeting process activates AKT (protein kinase B) and extracellular regulated kinases (ERK) 1/2 signaling pathways, thereby enhancing hypoxia inducible factor-1 alpha (HIF-1α) and VEGF expression (Di Bernardini et al., 2014; Zhou et al., 2014). Although, not significantly different, the slight increase of miR-21 after HVT (1.6-fold) in the present study, is consistent with findings from Baggish et al. (2011), in which circulating miR-21 was significantly up-regulated (1.9-fold) in human adults immediately following an incremental cycling step test. Based on other findings, one reason for the increased levels of miR-21 after HVT might be prolonged shear stress, the latter of which might not be present during HIIT. Shear stress has been shown to regulate the expression of miR-21 in endothelial cells (Weber et al., 2010). Nicoli et al. (2010) demonstrated that miR-21 plays a role in the integration of hemodynamics and VEGF signaling during angiogenesis (Nicoli et al., 2010; Weber et al., 2010). Although, HIIT produces higher blood flow velocities and therefore, higher shear stress than during HVT, the total time of exposure to shear stress might have been too short during HIIT in the current study.

miR-126, an endothelial specific miRNA, regulates vascular integrity and angiogenesis (Wang et al., 2008). miR-126 is induced by hypoxic stress (Truettner et al., 2013) and enhances the actions of VEGF and bFGF by repressing the expression of Sprouty-related protein-1, an inhibitor of angiogenic signaling (Wang et al., 2008). In the present study, miR-126 was not affected by the HIIT session. In previous studies with untrained human adults (42.0 ± 2.0 mL·kg^−1^·min^−1^), miR-126 was significantly down-regulated (1.53-fold) following a high intensity interval unit (10 × 2 min at 76% VO_2peak_; Radom-Aizik et al., 2010). The increase of miR-126 during (3.0-fold) and 60 min after (2.8-fold) HVT is in line with previous studies with human adults, although these studies investigated longer exercise durations than in the present investigation. Uhlemann et al. (2014) found that a 4 h cycling session at 70% of subjects' anaerobic threshold increased miR-126 plasma concentration with peak values occurring 30 min after the start (4.6-fold), which remained elevated up until the end of the training session (4.0 ± 0.8 -fold). In contrast to miR-21, Hergenreider et al. (2012) demonstrated that shear stress had neither an influence on miR-126 expression in Human Umbilical Vein Endothelial Cells, nor on the formation of miR-126 containing vesicles (Hergenreider et al., 2012).

Normally, the expression of VEGF mRNA is an intracellular process in endothelial cells. However, there are different mechanisms (exosomes, microparticles, apoptotic bodies) which can transport mRNA into the extracellular space and into the bloodstream (Valadi et al., 2007; Camussi et al., 2010). Previous studies with different purposes already measured circulating mRNA in the blood (Atamaniuk et al., 2008; Greiner et al., 2013; Tonevitsky et al., 2013). Therefore, the measured VEGF mRNA in the circulation might be an indirect indicator of an increased expression, which might reflect intercellular communication or cell damage and release of mRNA molecules. In the present study, a significant increase of VEGF mRNA was shown at d3, 0′, 60′, and 180′ post-exercise for HVT, and at d1 and 30′, 60′, and 180′ post-exercise for HIIT. So far, there are no comparable data for circulating VEGF mRNA. Hence, VEGF mRNA values from muscle and animal models must be consulted. Compared to the expression of VEGF mRNA in muscle biopsies, results of the present study reveal similar findings and time courses. Studies using muscle biopsies showed an increase in VEGF mRNA expression after knee extension as soon as 30 min post-exercise (Richardson et al., 2000; Gustafsson et al., 2002). Hiscock et al. (2003) measured a significant increase in VEGF mRNA during (1.5 h) and after (0′, 60′, 180′) a 3-h low-intensity training session (two-legged knee extension at 50% of peak workload). It can be assumed that both training stimuli in the present study increased the rate of expression of VEGF mRNA. Although the metabolic (pH, lactate) and mechanical stresses (blood pressure)—which are known to be potent stimuli to induce angiogenesis—were significantly higher during HIIT, it seems that the total time of exposure to the endothelium during HVT, might also be a positive stimuli for the expression of VEGF mRNA. This is supported by the result of the AUC for the post-exercise time points, which did not differ between HIIT and HVT. VEGF mRNA increased despite the significant increase of miR-16 after HVT and the fact that miR-16 has been suggested to be a potential suppressor of VEGF mRNA. However, the amount of activators on VEGF mRNA expression, like miR-21 and miR-126 might prevail, causing a significant increase of VEGF mRNA.

It has already been shown that miRNAs can enter the bloodstream under resting conditions and in response to tissue injury and other pathological conditions (Mitchell et al., 2008). Further, a specific regulation of miRNAs in response to physical activity has been highlighted in a number of studies (Radom-Aizik et al., 2010; Baggish et al., 2011). Mechanistic conclusions on the regulation of miRNAs in response to physical activity could not be verified in this study. A rapid up-regulation of miRNAs after short, high-intensity exercise is not a likely explanation for de novo transcription of miRNAs. Such transcription might be due to a post-transcriptional processing of pre-existing inactive pre- miRNAs. This mechanism has already been described in the literature for the miR-21 (Davis et al., 2008). Additionally, due to the high training status of the children in the present study (57.9 ± 9.4 ml·min^−1^·kg^−1^ peak oxygen uptake), one might expect a different regulation of circulating miRNAs in response to exercise in untrained children. Dawes et al. (2015) already reported a different miRNA expression between high- and low active mice (Dawes et al., 2015). Also in human adults differences in resting miRNA levels between different elite athletes (endurance vs. strength) and untrained controls were observed (Wardle et al., 2015).

## Conclusion

In line with previous studies, the present investigation show that acute exercise can alter circulating miRNA profiles. Our data demonstrate a volume-dependent regulation of vascular regulating miRNAs (miR-16, -21, -126) in children. However, the VEGF mRNA showed a duration and intensity-dependent regulation. So in the case of HVT, the amount of positive regulators (miR-21, miR-126) for an increase in circulating VEGF mRNA prevails. In the case of HIIT, VEGF mRNA is similarly increased in the absence of positive regulators, but also in the absence of a negative regulator (miR-16). Therefore, it might be speculated that VEGF as a “master regulator” of angiogenesis can be regulated differently. Future studies need to investigate the regulation of angiogenic processes under different exercise conditions and in different populations (young/old; trained/untrained; healthy/unhealthy) more in detail.

## Author contributions

All authors listed, have made substantial, direct and intellectual contribution to the work, and approved it for publication.

### Conflict of interest statement

The authors declare that the research was conducted in the absence of any commercial or financial relationships that could be construed as a potential conflict of interest.

## References

[B1] Altana V. Geretto M. Pulliero A. (2015). MicroRNAs and physical activity. Microrna 4, 74–85. 10.2174/2211536604666150813152450 26268469

[B2] Aoi W. Ichikawa H. Mune K. Tanimura Y. Mizushima K. Naito Y.. (2013). Muscle-enriched microRNA miR-486 decreases in circulation in response to exercise in young men. Front. Physiol. 4:80. 10.3389/fphys.2013.00080 23596423 PMC3622901

[B3] Arroyo J. D. Chevillet J. R. Kroh E. M. Ruf I. K. Pritchard C. C. Gibson D. F.. (2011). Argonaute2 complexes carry a population of circulating microRNAs independent of vesicles in human plasma. Proc. Natl. Acad. Sci. U.S.A. 108, 5003–5008. 10.1073/pnas.1019055108 21383194 PMC3064324

[B4] Atamaniuk J. Stuhlmeier K. M. Vidotto C. Tschan H. Dossenbach-Glaninger A. Mueller M. M. (2008). Effects of ultra-marathon on circulating DNA and mRNA expression of pro- and anti-apoptotic genes in mononuclear cells. Eur. J. Appl. Physiol. 104, 711–717. 10.1007/s00421-008-0827-2 18651163

[B5] Baggish A. L. Hale A. Weiner R. B. Lewis G. D. Systrom D. Wang F.. (2011). Dynamic regulation of circulating microRNA during acute exhaustive exercise and sustained aerobic exercise training. J. Physiol. 589, 3983–3994. 10.1113/jphysiol.2011.213363 21690193 PMC3179997

[B6] Baggish A. L. Park J. Min P. K. Isaacs S. Parker B. A. Thompson P. D.. (2014). Rapid upregulation and clearance of distinct circulating microRNAs after prolonged aerobic exercise. J. Appl. Physiol. 116, 522–531. 10.1152/japplphysiol.01141.2013 24436293 PMC3949215

[B7] Banzet S. Chennaoui M. Girard O. Racinais S. Drogou C. Chalabi H.. (2013). Changes in circulating microRNAs levels with exercise modality. J. Appl. Physiol. 115, 1237–1244. 10.1152/japplphysiol.00075.2013 23950168

[B8] Breil F. A. Weber S. N. Koller S. Hoppeler H. Vogt M. (2010). Block training periodization in alpine skiing: effects of 11-day HIT on VO_2max_ and performance. Eur. J. Appl. Physiol. 109, 1077–1086. 10.1007/s00421-010-1455-1 20364385

[B9] Camussi G. Deregibus M. C. Bruno S. Cantaluppi V. Biancone L. (2010). Exosomes/microvesicles as a mechanism of cell-to-cell communication. Kidney. Int. 78, 838–848. 10.1038/ki.2010.278 20703216

[B10] Caporali A. Emanueli C. (2011). MicroRNA regulation in angiogenesis. Vascul. Pharmacol. 55, 79–86. 10.1016/j.vph.2011.06.006 21777698

[B11] Chamorro-Jorganes A. Araldi E. Penalva L. O. Sandhu D. Fernandez-Hernando C. Suarez Y. (2011). MicroRNA-16 and microRNA-424 regulate cell-autonomous angiogenic functions in endothelial cells via targeting vascular endothelial growth factor receptor-2 and fibroblast growth factor receptor-1. Arterioscler. Thromb. Vasc. Biol. 31, 2595–2606. 10.1161/ATVBAHA.111.236521 21885851 PMC3226744

[B12] Davidsen P. K. Gallagher I. J. Hartman J. W. Tarnopolsky M. A. Dela F. Helge J. W.. (2011). High responders to resistance exercise training demonstrate differential regulation of skeletal muscle microRNA expression. J. Appl. Physiol. 110, 309–317. 10.1152/japplphysiol.00901.2010 21030674

[B13] Davidson-Moncada J. Papavasiliou F. N. Tam W. (2010). MicroRNAs of the immune system. roles in inflammation and cancer. Ann. N.Y. Acad. Sci. 1183, 183–194. 10.1111/j.1749-6632.2009.05121.x 20146715 PMC2876712

[B14] Davis B. N. Hilyard A. C. Lagna G. Hata A. (2008). SMAD proteins control DROSHA-mediated microRNA maturation. Nature 454, 56–61. 10.1038/nature07086 18548003 PMC2653422

[B15] Dawes M. Kochan K. J. Riggs P. K. Timothy Lightfoot J. (2015). Differential miRNA expression in inherently high- and low-active inbred mice. Physiol. Rep. 3, e12469–e12482. 10.14814/phy2.12469 26229004 PMC4552544

[B16] Di Bernardini E. Campagnolo P. Margariti A. Zampetaki A. Karamariti E. Hu Y.. (2014). Endothelial lineage differentiation from induced pluripotent stem cells is regulated by microRNA-21 and transforming growth factor β2 (TGF-β2) pathways. J. Biol. Chem. 289, 3383–3393. 10.1074/jbc.M113.495531 24356956 PMC3916541

[B17] Di Prampero P. E. Ferretti G. (1999). The energetics of anaerobic muscle metabolism: a reappraisal of older and recent concepts. Respir. Physiol. 118, 103–115. 10.1016/S0034-5687(99)00083-3 10647856

[B18] Egginton S. (2009). Invited review: activity-induced angiogenesis. Pflugers Arch. 457, 963–977. 10.1007/s00424-008-0563-9 18704490

[B19] Engel F. Härtel S. Wagner M. O. Strahler J. Bös K. Sperlich B. (2014). Hormonal, metabolic, and cardiorespiratory responses of young and adult athletes to a single session of high-intensity cycle exercise. Pediatr. Exerc. Sci. 26, 485–494. 10.1123/pes.2013-0152 25050695

[B20] Faude O. Meyer T. Scharhag J. Weins F. Urhausen A. Kindermann W.. (2008). Volume vs. intensity in the training of competitive swimmers. Int. J. Sports. Med. 29, 906–912. 10.1055/s-2008-1038377 18418808

[B21] Fernandes T. Magalhaes F. C. Roque F. R. Phillips M. I. Oliveira E. M. (2012). Exercise training prevents the microvascular rarefaction in hypertension balancing angiogenic and apoptotic factors. role of micrornas-16, -21, and -126. Hypertension 59, 513–520. 10.1161/HYPERTENSIONAHA.111.185801 22215713

[B22] Fichtlscherer S. Zeiher A. M. Dimmeler S. Sessa W. C. (2011). Circulating MicroRNAs. Biomarkers or mediators of cardiovascular diseases? Arterioscler. Thromb. Vasc. Biol. 31, 2383–2390. 10.1161/ATVBAHA.111.226696 22011751

[B23] Fish J. E. Srivastava D. (2009). MicroRNAs. Opening a new vein in angiogenesis research. Sci. Signal. 2:pe1. 10.1126/scisignal.252pe1 19126861 PMC2680274

[B24] Gibala M. (2009). Molecular responses to high-intensity interval exercise. Appl. Physiol. Nutr. Metab. 34, 428–432. 10.1139/H09-046 19448710

[B25] Gibala M. J. McGee S. L. Garnham A. P. Howlett K. F. Snow R. J. Hargreaves M. (2009). Brief intense interval exercise activates AMPK and p38 MAPK signaling and increases the expression of PGC-1alpha in human skeletal muscle. J. Appl. Physiol. 106, 929–934. 10.1152/japplphysiol.90880.2008 19112161

[B26] Greiner H. M. Horn P. S. Holland K. Collins J. Hershey A. D. Glauser T. A. (2013). mRNA blood expression patterns in new-onset idiopathic pediatric epilepsy. Epilepsia 54, 272–279. 10.1111/epi.12016 23167847 PMC3566372

[B27] Gustafsson T. Knutsson A. Puntschart A. Kaijser L. Nordqvist A. C. Sundberg C. J.. (2002). Increased expression of vascular endothelial growth factor in human skeletal muscle in response to short-term one-legged exercise training. Pflugers Arch. 444, 752–759. 10.1007/s00424-002-0845-6 12355175

[B28] He L. Hannon G. J. (2004). MicroRNAs. Small RNAs with a big role in gene regulation. Nat. Rev. Genet. 5, 522–531. 10.1038/nrg1379 15211354

[B29] Hergenreider E. Heydt S. Tréguer K. Boettger T. Horrevoets A. J. G. Zeiher A. M.. (2012). Atheroprotective communication between endothelial cells and smooth muscle cells through miRNAs. Nat. Cell. Biol. 14, 249–256. 10.1038/ncb2441 22327366

[B30] Hiscock N. Fischer C. P. Pilegaard H. Pedersen B. K. (2003). Vascular endothelial growth factor mRNA expression and arteriovenous balance in response to prolonged, submaximal exercise in humans. Am. J. Physiol. Heart. Circ. Physiol. 285, H1759–H1763. 10.1152/ajpheart.00150.2003 12763746

[B31] Jansen F. Yang X. Hoelscher M. Cattelan A. Schmitz T. Proebsting S.. (2013). Endothelial microparticle-mediated transfer of MicroRNA-126 promotes vascular endothelial cell repair via SPRED1 and is abrogated in glucose-damaged endothelial microparticles. Circulation 128, 2026–2038. 10.1161/CIRCULATIONAHA.113.001720 24014835

[B32] Lewis B. P. Burge C. B. Bartel D. P. (2005). Conserved seed pairing, often flanked by adenosines, indicates that thousands of human genes are microRNA targets. Cell 120, 15–20. 10.1016/j.cell.2004.12.035 15652477

[B33] Livak K. J. Schmittgen T. D. (2001). Analysis of relative gene expression data using real-time quantitative PCR and the 2(-Delta Delta C(T)) Method. Methods 25, 402–408. 10.1006/meth.2001.1262 11846609

[B34] Mitchell P. S. Parkin R. K. Kroh E. M. Fritz B. R. Wyman S. K. Pogosova-Agadjanyan E. L.. (2008). Circulating microRNAs as stable blood-based markers for cancer detection. Proc. Natl. Acad. Sci. U.S.A. 105, 10513–10518. 10.1073/pnas.0804549105 18663219 PMC2492472

[B35] Nicoli S. Standley C. Walker P. Hurlstone A. Fogarty K. E. Lawson N. D. (2010). MicroRNA-mediated integration of haemodynamics and Vegf signalling during angiogenesis. Nature 464, 1196–1200. 10.1038/nature08889 20364122 PMC2914488

[B36] Nielsen S. Scheele C. Yfanti C. Akerstrom T. Nielsen A. R. Pedersen B. K.. (2010). Muscle specific microRNAs are regulated by endurance exercise in human skeletal muscle. J. Physiol. 588, 4029–4037. 10.1113/jphysiol.2010.189860 20724368 PMC3000590

[B37] Pritchett K. Bishop P. Pritchett R. Green M. Katica C. (2009). Acute effects of chocolate milk and a commercial recovery beverage on postexercise recovery indices and endurance cycling performance. Appl. Physiol. Nutr. Metab. 34, 1017–1022. 10.1139/H09-104 20029509

[B38] Proctor D. N. Sinning W. E. Walro J. M. Sieck G. C. Lemon P. W. (1995). Oxidative capacity of human muscle fiber types: effects of age and training status. J. Appl. Physiol. 78, 2033–2038. 10.1249/00005768-199505001-00247 7665396

[B39] Quintavalle C. Garofalo M. Croce C. M. Condorelli G. (2011). “ApoptomiRs” in vascular cells. Their role in physiological and pathological angiogenesis. Vascul. Pharmacol. 55, 87–91. 10.1016/j.vph.2011.07.004 21798370

[B40] Radom-Aizik S. Zaldivar F. Jr. Oliver S. Galassetti P. Cooper D. M. (2010). Evidence for microRNA involvement in exercise-associated neutrophil gene expression changes. J. Appl. Physiol. 109, 252–261. 10.1152/japplphysiol.01291.2009 20110541 PMC2904199

[B41] Richardson R. S. Wagner H. Mudaliar S. R. Saucedo E. Henry R. Wagner P. D. (2000). Exercise adaptation attenuates VEGF gene expression in human skeletal muscle. Am. J. Physiol. Heart. Circ. Physiol. 279, H772–H778. 10924077 10.1152/ajpheart.2000.279.2.H772

[B42] Robison E. H. Mondala T. S. Williams A. R. Head S. R. Salomon D. R. Kurian S. M. (2009). Whole genome transcript profiling from fingerstick blood samples: a comparison and feasibility study. BMC Genomics 10:617. 10.1186/1471-2164-10-617 20017944 PMC2811129

[B43] Ryan N. A. Zwetsloot K. A. Westerkamp L. M. Hickner R. C. Pofahl W. E. Gavin T. P. (2006). Lower skeletal muscle capillarization and VEGF expression in aged vs. young men. J. Appl. Physiol. 100, 178–185. 10.1152/japplphysiol.00827.2005 16166239

[B44] Sandbakk Ø. Sandbakk S. B. Ettema G. Welde B. (2013). Effects of intensity and duration in aerobic high-intensity interval training in highly trained junior cross-country skiers. J. Strength. Cond. Res. 27, 1974–1980. 10.1519/JSC.0b013e3182752f08 23037620

[B45] Scott C. B. (2005). Contribution of anaerobic energy expenditure to whole body thermogenesis. Nutr. Metab. 2:14. 10.1186/1743-7075-2-14 15958171 PMC1182393

[B46] Scott C. B. Kemp R. B. (2005). Direct and indirect calorimetry of lactate oxidation: implications for whole-body energy expenditure. J. Sports. Sci. 23, 15–19. 10.1080/02640410410001716760 15841591

[B47] Scott C. B. Littlefield N. D. Chason J. D. Bunker M. P. Asselin E. M. (2006). Differences in oxygen uptake but equivalent energy expenditure between a brief bout of cycling and running. Nutr. Metab. 3:1. 10.1186/1743-7075-3-1 16390548 PMC1334197

[B48] Sperlich B. De Marées M. Koehler K. Linville J. Holmberg H. C. Mester J. (2011). Effects of 5 weeks of high-intensity interval training vs. volume training in 14-year-old soccer players. J. Strength. Cond. Res. 25, 1271–1278. 10.1519/JSC.0b013e3181d67c38 21490513

[B49] Suárez Y. Sessa W. C. (2009). MicroRNAs as novel regulators of angiogenesis. Circ. Res. 104, 442–454. 10.1161/CIRCRESAHA.108.191270 19246688 PMC2760389

[B50] Tonevitsky A. G. Maltseva D. V. Abbasi A. Samatov T. R. Sakharov D. A. Shkurnikov M. U.. (2013). Dynamically regulated miRNA-mRNA networks revealed by exercise. BMC Physiol. 13:9. 10.1186/1472-6793-13-9 24219008 PMC3681679

[B51] Triozzi P. L. Achberger S. Aldrich W. Singh A. D. Grane R. Borden E. C. (2012). The association of blood angioregulatory microRNA levels with circulating endothelial cells and angiogenic proteins in patients receiving dacarbazine and interferon. J. Transl. Med. 10:241. 10.1186/1479-5876-10-241 23217102 PMC3573971

[B52] Truettner J. S. Katyshev V. Esen-Bilgin N. Dietrich W. D. Dore-Duffy P. (2013). Hypoxia alters MicroRNA expression in rat cortical pericytes. Microrna 2, 32–44. 10.2174/2211536611302010005 24883265 PMC4039645

[B53] Uhlemann M. Möbius-Winkler S. Fikenzer S. Adam J. Redlich M. Möhlenkamp S.. (2014). Circulating microRNA-126 increases after different forms of endurance exercise in healthy adults. Eur. J. Prev. Cardiol. 21, 484–491. 10.1177/2047487312467902 23150891

[B54] Urbich C. Kuehbacher A. Dimmeler S. (2008). Role of microRNAs in vascular diseases, inflammation, and angiogenesis. Cardiovasc. Res. 79, 581–588. 10.1093/cvr/cvn156 18550634

[B55] Valadi H. Ekström K. Bossios A. Sjöstrand M. Lee J. J. Lötvall J. O. (2007). Exosome-mediated transfer of mRNAs and microRNAs is a novel mechanism of genetic exchange between cells. Nat. Cell. Biol. 9, 654–659. 10.1038/ncb1596 17486113

[B56] Wahl P. Jansen F. Achtzehn S. Schmitz T. Bloch W. Mester J.. (2014). Effects of high intensity training and high volume training on endothelial microparticles and angiogenic growth factors. PLoS ONE 9:e96024. 10.1371/journal.pone.0096024 24770423 PMC4000202

[B57] Wahl P. Zinner C. Achtzehn S. Behringer M. Bloch W. Mester J. (2011). Effects of acid-base balance and high or low intensity exercise on VEGF and bFGF. Eur. J. Appl. Physiol. 111, 1405–1413. 10.1007/s00421-010-1767-1 21161264

[B58] Wahl P. Zinner C. Grosskopf C. Rossmann R. Bloch W. Mester J. (2013). Passive recovery is superior to active recovery during a high-intensity shock microcycle. J. Strength. Cond. Res. 27, 1384–1393. 10.1519/JSC.0b013e3182653cfa 22744298

[B59] Wang R. Li N. Zhang Y. Ran Y. Pu J. (2011). Circulating microRNAs are promising novel biomarkers of acute myocardial infarction. Intern. Med. 50, 1789–1795. 10.2169/internalmedicine.50.5129 21881276

[B60] Wang S. Aurora A. B. Johnson B. A. Qi X. McAnally J. Hill J. A.. (2008). The endothelial-specific MicroRNA miR-126 governs vascular integrity and angiogenesis. Dev. Cell 15, 261–271. 10.1016/j.devcel.2008.07.002 18694565 PMC2685763

[B61] Wardle S. L. Bailey M. E. S. Kilikevicius A. Malkova D. Wilson R. H. Venckunas T.. (2015). Plasma microRNA levels differ between endurance and strength athletes. PLoS ONE 10:e0122107. 10.1371/journal.pone.0122107 25881132 PMC4400105

[B62] Weber M. Baker M. B. Moore J. P. Searles C. D. (2010). MiR-21 is induced in endothelial cells by shear stress and modulates apoptosis and eNOS activity. Biochem. Biophys. Res. Commun. 393, 643–648. 10.1016/j.bbrc.2010.02.045 20153722 PMC3717387

[B63] Wehmeier U. F. Hilberg T. (2014). Capillary earlobe blood may be used for RNA isolation, gene expression assays and microRNA quantification. Mol. Med. Rep. 9, 211–216. 10.3892/mmr.2013.1779 24213018

[B64] Xu T. Liu Q. Yao J. Dai Y. Wang H. Xiao J. (2015). Circulating microRNAs in response to exercise. Scand. J. Med. Sci. Sports. 25, e149–e154. 10.1111/sms.12421 25648616

[B65] Yasuda S. Arii S. Mori A. Isobe N. Yang W. Oe H.. (2004). Hexokinase II and VEGF expression in liver tumors: correlation with hypoxia-inducible factor 1 alpha and its significance. J. Hepatol. 40, 117–123. 10.1016/S0168-8278(03)00503-8 14672622

[B66] Zampetaki A. Willeit P. Drozdov I. Kiechl S. Mayr M. (2011). Profiling of circulating microRNAs: from single biomarkers to re-wired networks. Cardiovasc. Res. 93, 555–562. 10.1093/cvr/cvr266 22028337 PMC3291086

[B67] Zhou Y. Yang F. Chen T. Wu Y. Yang M. Zhu J.. (2014). An updated view on the differentiation of stem cells into endothelial cells. Sci. China Life Sci. 57, 763–773. 10.1007/s11427-014-4712-4 25104448

[B68] Zhu L.-J. Altmann S. W. (2005). mRNA and 18S-RNA coapplication-reverse transcription for quantitative gene expression analysis. Anal. Biochem. 345, 102–109. 10.1016/j.ab.2005.07.028 16139233

[B69] Zinner C. Wahl P. Achtzehn S. Reed J. L. Mester J. (2014). Acute hormonal responses before and after 2 weeks of HIT in well-trained junior triathletes. Int. J. Sports. Med. 35, 316–322. 10.1055/s-0033-1353141 24081622

